# Prevention of Legionnaires’ Disease in the 21st Century by Advancing Science and Public Health Practice

**DOI:** 10.3201/eid2311.171429

**Published:** 2017-11

**Authors:** Ruth L. Berkelman, Amy Pruden

**Affiliations:** Emory University, Atlanta, Georgia, USA (R.L. Berkelman);; Virginia Polytechnic Institute and State University, Blacksburg, Virginia, USA (A. Pruden)

**Keywords:** Legionnaires’ disease, legionellosis, Legionella, Legionella pneumophila, bacteria, prevention, policy, Safe Water Drinking Act, public health practice, water management programs, water systems, validation, nontuberculous mycobacteria, tuberculosis and other mycobacteria

A dramatic shift in emphasis by public health officials from detection, investigation, and control of outbreaks of Legionnaires’ disease to primary prevention strategies is underway in the United States. Expansion of primary prevention efforts is occurring with remarkable speed and is marked by new public health guidance and policy aimed at ensuring adequacy of water systems management (*1**–**5*). Professional societies are also providing more rigorous and detailed guidance. The American Society of Heating, Refrigerating and Air-Conditioning Engineers approved an industry standard in 2015 and established minimum management requirements to reduce the risk for infection with *Legionella* spp., the bacteria that cause Legionnaires’ disease, in building water systems (*6*). In 2015, the American Industrial Hygiene Society also released specific guidance on control of *Legionella* spp. in engineered water systems and focused on validation of water management efforts and testing water for viable *Legionella* spp. counts as the key tool to validate effectiveness of these efforts (*7*). In June 2017, the Centers for Medicaid and Medicare Services issued a requirement to reduce risk for infection with *Legionella* spp. in healthcare facilities (*8*).

This sea of change in *Legionella* spp. program focus in public health is occurring because of a convergence of factors. The >5-fold increase in cases of disease caused by *Legionella* spp. during 2000−2015 has mandated a substantive public health response. The increase has been widely recognized as likely to continue unabated in the absence of more effective prevention strategies. Recent large outbreaks, such as those in the Bronx, New York, USA (*9*), have been critical in garnering public attention and generating momentum for more robust prevention efforts. The fact that analysis by the Centers for Disease Control and Prevention indicated that only 4% of Legionnaires’ disease cases were outbreak-associated has further illustrated limitations of outbreak detection and control as the primary prevention tool (*1*). Supported by the Alfred P. Sloan Foundation, the first public health conference on *Legionella* spp. in 25 years identified gaping holes in our knowledge of prevention of infections with *Legionella* spp. and indicated the need for robust federally sponsored research (*10*).

Numerous factors are contributing to the increase in incidence of Legionnaires’ disease. Increasing numbers of persons are at increased risk because of aging of the population, greater use of immunosuppressant drugs, and higher prevalence of comorbid conditions. Changing environmental conditions are also facilitating human exposure to aerosolized water containing *Legionella* spp. There is a growing dependence on heating, ventilation, and cooling systems, as well as increased complexity of indoor plumbing systems in large buildings, which have a labyrinth of water lines and features ranging from hundreds of showerheads in rooms along lengthy corridors to spas and indoor decorative fountains. Potential new sources, such as street cleaning machines reported by Valero Munoz et al. in this issue (*11*), continue to be identified. Inadequate maintenance of public water supplies might result in increased risk for contamination of building water systems and other water devices or equipment. Also in this issue, Lapierre et al. suggest potential new ways water systems can become contaminated; this article describes the potential for cross-contamination of cooling towers (*12*).

Healthcare providers play an essential role in diagnosing disease; diagnosis assists with targeting antimicrobial drug therapy, as well as playing a critical role in public health surveillance. The urine antigen test identifies only *L. pneumophila* serogroup 1, and its use minimizes the role of other *Legionella* subtypes and species (*13*). Respiratory diagnostic panels are increasing in number and need to include the capacity to identify all species of *Legionella *(*14*)*.*

Ideally, a combined approach of surveillance and improved fundamental understanding of the factors triggering proliferation of *Legionella* spp. will inform optimal engineering design and industrial hygiene practice. *Legionella* spp. are known to have a symbiotic relationship with certain protozoa that graze on drinking water biofilms, and infection of these hosts can enhance virulence of the bacteria. Premise (i.e., building) plumbing can be a reservoir for pathogens such as *Legionella* spp., even in the presence of high disinfectant residuals (e.g., chlorine), and different microbes display distinct responses to various disinfectants, pipe materials, and water ages and their interactions.

Water-conserving technologies, such as reuse practices and high-volume water storage, can result in high water age, diminished protective disinfectant residuals, and degraded water quality. Low-flow velocities of certain water-conserving fixtures, now popular in many “green” buildings, might fail to adequately flush out biofilms and other particulates conducive to *Legionella* spp. growth. Similarly, reducing water heater temperatures may play a role in saving energy and reducing scalding, but if these benefits are found to be marginal relative to the potential to spread disease, these technologies should be reevaluated. Thus, as we as a society strive for new water- and energy-conserving building designs, the potential to stimulate *Legionella* spp. growth warrants serious consideration.

Respiratory pathogens other than *Legionella* spp., including nontuberculous mycobacteria (NTM) and *Pseudomonas* spp., are also found in engineered water systems. The prevalence of cases of infection with NTM is increasing dramatically and resulting in the hospitalizations of thousands of patients each year and months to years of multiple antimicrobial drug use (*15*). Whether current measures for prevention of *Legionella* spp. amplification will control other waterborne pathogens, such as NTM, is unclear. The US Environmental Protection Agency Safe Drinking Water Act, which has been in effect since the 1970s, is geared toward risk for fecal pathogens being ingested. This act does not take into account pathogens that adhere to biofilms in pipes and grow in various conditions.

Collaboration is needed across scientific disciplines and agencies to fill gaps in our collective knowledge of modern water pathogens, weaknesses in our physical water infrastructure, and inadequacies of our existing diagnostic and regulatory approaches to effectively identify and control disease risks. *Legionella* spp. illuminate many of the challenges posed by water pathogens in the 21st century. With a growing national interest in a safe water supply, it is an opportune time for public health and medicine to come together with industrial hygiene, engineering, microbiology, and environmental protection to understand the problems and ensure effective risk management of *Legionella* spp. and other pathogens inhabiting our water systems.
